# Retropharyngeal Abscess: Patterns of Deep Neck Space Extension and Clinical Findings From 11 Adult Cases

**DOI:** 10.7759/cureus.100942

**Published:** 2026-01-06

**Authors:** Giorgos Sideris, Eleni Vasileiou, Ioannis E Papachristos, Sotirios Karamagkiolas, Petros V Vlastarakos, Nikolaos Papadimitriou, Alexander Delides

**Affiliations:** 1 2nd ENT Department, Attikon University Hospital, National and Kapodistrian University of Athens, Athens, GRC; 2 Department of Radiology, Biotypos Diagnostic Center, Athens, GRC

**Keywords:** adult, cervical spondylodiscitis, danger space, deep neck space infection, parapharyngeal space, prevertebral space, retropharyngeal abscess, surgical drainage

## Abstract

Introduction: Retropharyngeal abscess (RPA) in adults is rare but associated with significant morbidity due to rapid extension into deep neck spaces and the risk of life-threatening complications. Early diagnosis and intervention remain essential, yet management strategies continue to be debated. This study aims to analyze epidemiological, clinical, imaging, and management-related characteristics of adult RPA cases and identify factors associated with disease severity, spread, and outcomes.

Methods: A retrospective chart review was conducted at the Otolaryngology Department of a tertiary university hospital between January 2016 and October 2025. Adult patients with clinically and radiologically confirmed RPA were included. Data were collected on demographics, comorbidities, clinical presentation, microbiological findings, imaging results, airway status, management strategies, and outcomes, along with laboratory parameters including WBC and CRP levels.

Results: Eleven adults (seven males, four females; mean age 53.5 ± 15.4 years) were included. Mean WBC was 16.5 ± 3.11 ×10³/µL and mean CRP was 191 ± 110 mg/L. The most common symptoms were odynophagia and fever. Nine patients (81.8%) demonstrated radiological extension into deep neck spaces, most frequently the parapharyngeal and prevertebral spaces. Surgical drainage was performed in 10 patients; eight underwent transoral drainage, two required combined approaches, and two required tracheostomy. Staphylococcus species were the most common isolates. Mean hospitalization was 14.2 days (range 4-42). All patients survived.

Conclusions: Adult RPA frequently presents with extensive spread beyond the retropharyngeal space, owing to the anatomical continuity of this compartment, which facilitates rapid extension to the parapharyngeal, prevertebral, and mediastinal regions. Contrast-enhanced CT is essential for defining disease extent. Prompt airway evaluation and early broad-spectrum antibiotic therapy combined with surgical drainage remains the cornerstone of management, contributing to favorable outcomes.

## Introduction

Retropharyngeal abscess (RPA) is a deep neck space infection characterized by a collection of pus within the retropharyngeal space, located between the buccopharyngeal fascia and the alar fascia [1,2]. RPA is considered an acute deep neck space infection; prolonged or indolent presentations typically reflect delayed diagnosis or association with underlying chronic infectious processes (such as tuberculosis, actinomycosis, vertebral osteomyelitis, or spondylodiscitis), rather than a distinct chronic disease entity. Although more common in children due to the presence of retropharyngeal lymph nodes, RPA in adults is uncommon but significantly more severe [3,4]. If left untreated or inadequately managed, it may progress to life-threatening complications such as airway obstruction, aspiration of abscess contents, sepsis, internal jugular vein thrombosis, carotid sheath erosion, epidural abscess, and downward spread into the danger space leading to mediastinitis [1,5-8].

Adult patients typically present with odynophagia, dysphagia, neck swelling and/or stiffness, fever, malaise, and various degrees of dyspnea or stridor. Posterior pharyngeal wall bulging may be observed, although clinical findings are often subtle and nonspecific, making early diagnosis difficult. Imaging techniques - including contrast-enhanced computed tomography (CT) and magnetic resonance imaging (MRI) - are essential for confirming the diagnosis and assessing the extent of infection [9]. 

The management of RPA remains a subject of debate. While broad-spectrum intravenous antibiotics are universally required, the indications, timing, and optimal surgical approach for drainage in adults continue to be controversial [10].

The present retrospective study analyzes a case series of adult patients diagnosed with RPA. The primary objective is to provide a descriptive assessment of radiological patterns of spread into adjacent deep neck spaces and to examine associated management strategies and clinical outcomes in an adult-only population.

## Materials and methods

Study design and setting

This study was designed as a retrospective observational chart review conducted at the 2nd Otolaryngology Department of “Attikon” University Hospital, a tertiary referral center. Medical records from January 2016 to October 2025 were reviewed. The study protocol was approved by the Institutional Review Board and Ethics Committee of “Attikon” University Hospital (IRB approval number 749/19-11-2025) and was conducted in accordance with the principles of the Declaration of Helsinki. Due to the retrospective nature of the study, the requirement for informed consent was waived.

Study population

Adult patients (≥18 years) diagnosed with RPA during the study period were considered eligible. The diagnosis of RPA was established based on a combination of clinical presentation, physical examination findings, and radiological confirmation on contrast-enhanced CT. Only patients with complete medical records allowing full data extraction were included.

Inclusion and exclusion criteria

Inclusion criteria comprised: a) clinical presentation consistent with deep neck space infection (e.g., dysphagia, odynophagia, fever, neck pain, or airway symptoms), b) radiological confirmation of RPA on contrast-enhanced CT, with or without extension to adjacent deep neck spaces, and c) availability of complete medical records including laboratory data, imaging findings, treatment details, and clinical outcomes.

Patients were excluded if they were younger than 18 years, lacked radiological confirmation of RPA, or had incomplete or insufficient medical records for data extraction. In addition, cases of cervical infections or abscesses without retropharyngeal involvement were excluded. Recurrent hospitalizations of the same patient were considered a single case, with only the initial presentation included in the analysis.

Data collection

Data were retrospectively extracted from electronic and paper medical records using a standardized data collection form. Recorded variables included demographic characteristics (age, sex), clinical presentation and symptomatology, suspected etiology, comorbidities, the results of two critical lab tests: white blood cell (WBC) count and C-reactive protein (CRP), and imaging findings detailing the extent of RPA and involvement of adjacent deep neck spaces. Additional data included airway status and need for airway intervention, microbiological culture and sensitivity results, treatment strategies (antibiotic therapy, surgical drainage, surgical approach, type of anesthesia, and need for tracheostomy), length of hospitalization, complications, and clinical outcomes.

Treatment protocol

All patients received empiric broad-spectrum intravenous antibiotic therapy upon admission, targeting aerobic and anaerobic gram-positive and gram-negative organisms, in accordance with institutional protocols. Antibiotic regimens were subsequently adjusted based on microbiological culture and sensitivity results. Decisions regarding surgical drainage and choice of surgical approach (transoral, transcervical, or combined) were not based on a predefined protocol but were individualized based on clinical severity and imaging findings. Airway assessment was performed in all patients upon admission. Endotracheal intubation under direct laryngoscopy was used in patients requiring general anesthesia. Video-laryngoscopy was not routinely available or employed during the study period. Tracheostomy was reserved for cases with significant airway compromise or anticipated difficulty with safe endotracheal intubation.

Statistical analysis

Descriptive statistical analysis was performed using standard methods. Continuous variables were expressed as mean ± standard deviation and ranges, while categorical variables were presented as absolute numbers and percentages. Given the small sample size, no inferential statistical analysis was performed.

## Results

Eleven adult patients were included in this study. The mean total WBC count at presentation was 16.5 ± 3.11 × 10³/µL (95% CI: 14.66-18.34), while the mean CRP level was 191 ± 110 mg/L (95% CI: 126-256). Odynophagia and fever were the most frequently reported symptoms, followed by neck swelling, lumbar pain, hoarseness, and stridor. No patient had received oral antibiotics prior to admission. Only one patient had diabetes mellitus or another form of immunosuppression, and a single case was associated with foreign body ingestion, while the remaining 10 cases were considered spontaneous (Table 1).

**Table 1:** Demographic, laboratory, and clinical characteristics of adult patients with RPA. Values are presented as mean ± standard deviation or number (percentage), as appropriate. Reference ranges: WBC: 4.0–10.0 ×10³/µL; CRP: <5 mg/L. RPA: retropharyngeal abscess.

No of patients		11
	Male	7 (63.6%)
	Female	4 (36.4%)
Mean age (years)		53,5 ± 15,4
	95% CI	44-62
WBC (× 10³/µL)		16.5 ± 3.11
	95% CI	14.66-18.34
CRP (mg/L)		191 ± 110
	95% CI	126-256
Comorbidities (any)		4 (36.4%)
	Hypertension	3 (27.3%)
	Diebetes Mellitus	1 (9.1%)

**Figure 1:**
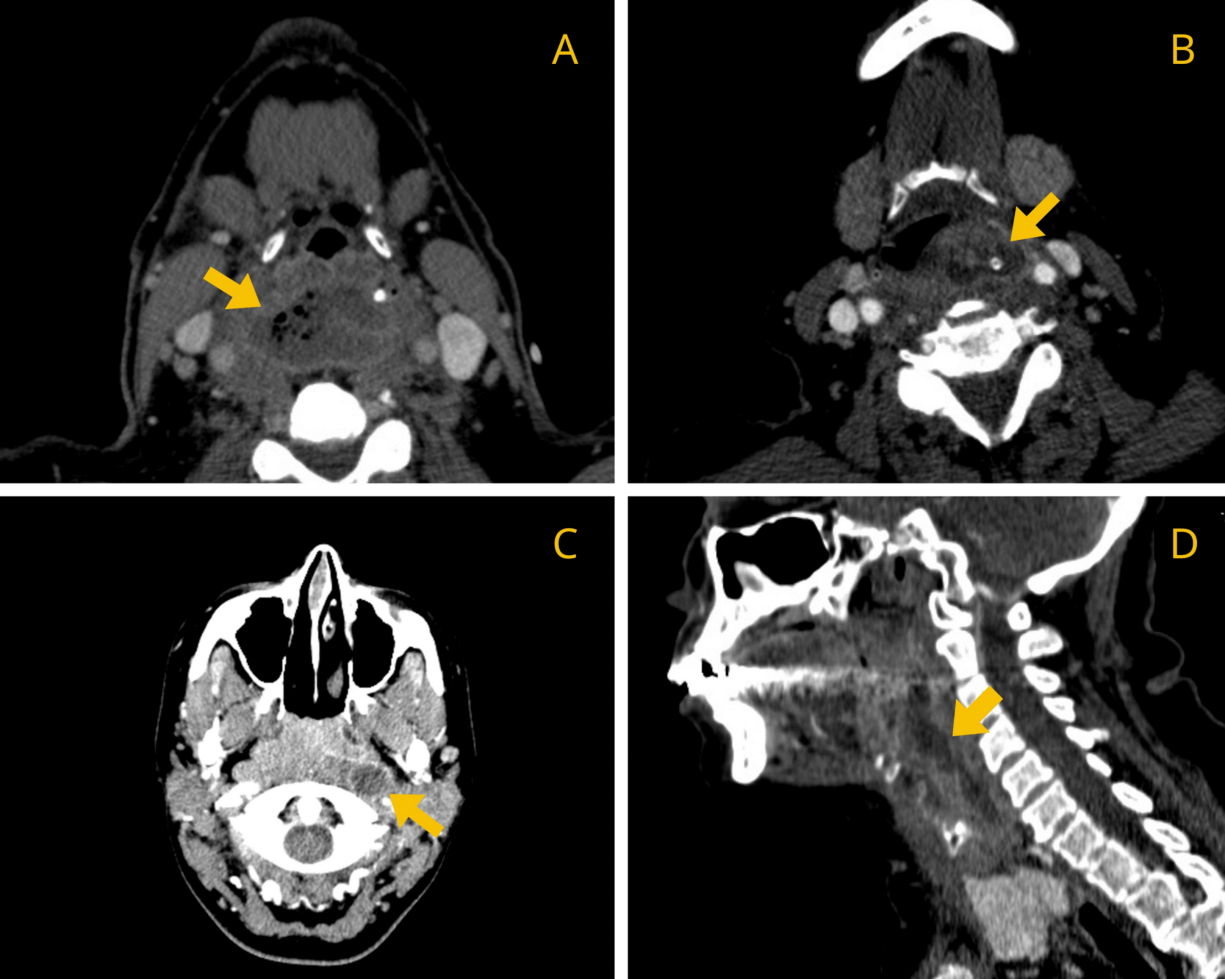
Contrast-enhanced CT images of patients 1 (A), 3 (B), 6 (C), and 9 (D) demonstrating RPA (yellow arrow). RPA: retropharyngeal abscess.

**Figure 2:**
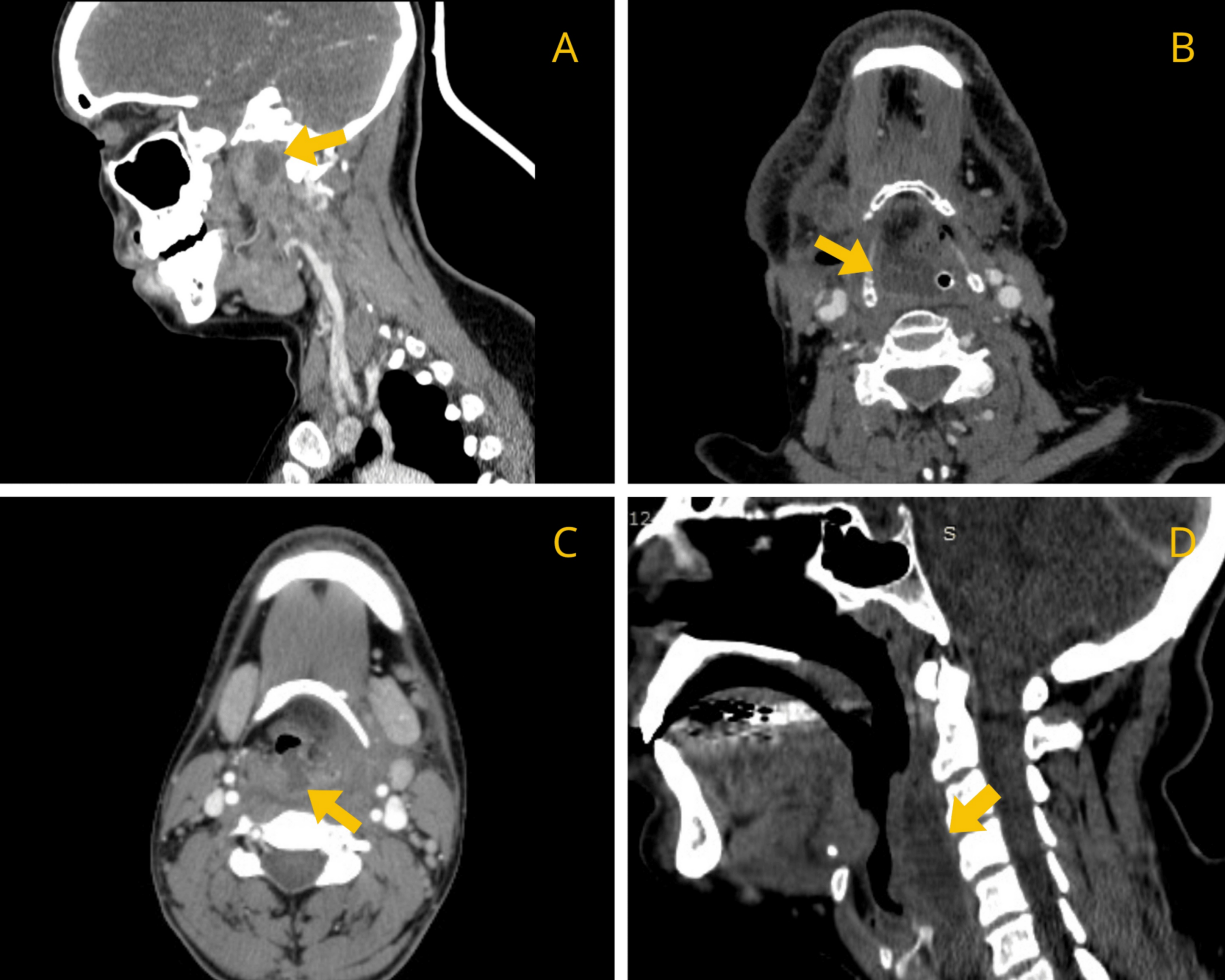
Contrast-enhanced CT images of patients 2 (A), 4 (B), 5 (C), and 11 (D) demonstrating RPA (yellow arrow). RPA: retropharyngeal abscess.

**Figure 3:**
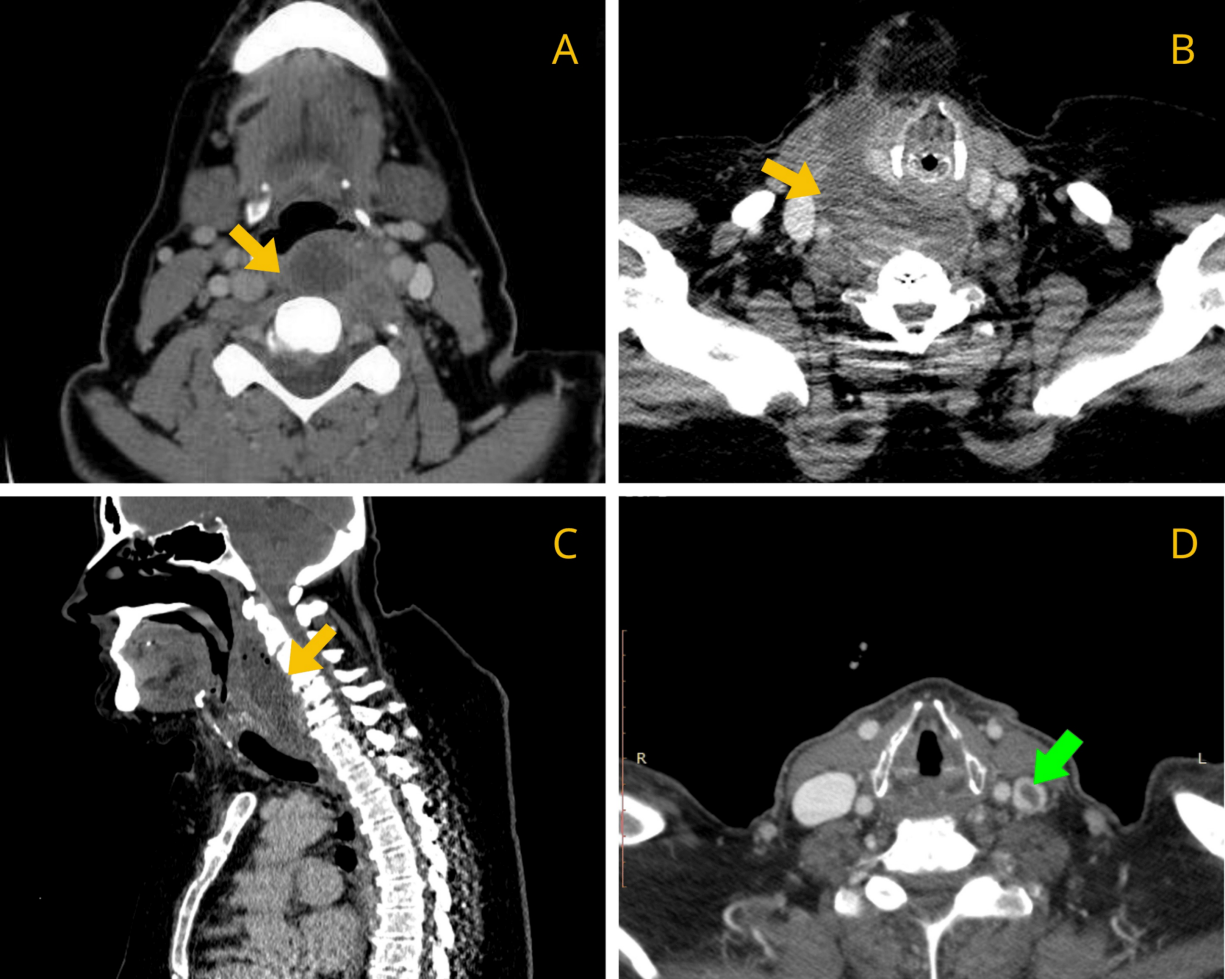
Contrast-enhanced CT images of patients 7 (A), 8 (B-C) and 10 (D). (A) Abscess extending to the prevertebral space (yellow arrow), (B-C) Abscess extending to the prevertebral space, posterior pharyngeal space, danger space (yellow arrow), (D) Left jugular vein thrombosis (green arrow).

A contrast-enhanced CT scan was performed in all cases to confirm diagnosis and assess the extent of involvement. Radiological evaluation demonstrated that nine patients (81.8%) exhibited RPA with extension into additional deep neck or other anatomical spaces. Only two patients had isolated RPAs without documented spread. Cervical spondylodiscitis was identified in two cases (Figures 1-3).

Ten patients underwent surgical drainage of the abscess. Seven procedures were performed under general anesthesia and three under local anesthesia. One patient experienced spontaneous intraoral drainage without the need for operative intervention. The transoral approach was used for drainage of RPA in eight patients while a combined transoral and transcervical approach was required in two cases. Despite the frequent use of general anesthesia, definitive airway protection with tracheostomy was required in only two patients, both presenting with marked airway compromise related to extensive supraglottic or parapharyngeal involvement. Pus cultures were obtained from all patients. Microbial growth was identified in nine samples, with Staphylococcus species representing the most frequently isolated organisms. In three cultures, multiple pathogens were isolated. In two cases, no microbial growth was detected, likely reflecting prior empirical antibiotic administration or limitations of culture sensitivity in deep neck space infections. The mean duration of hospitalization was 14.2 days (range: 4-42 days), reflecting variability in disease severity, extent of spread, and required interventions. All patients survived (Table 2).

**Table 2:** Summary of clinical, imaging and surgical characteristics of 11 adult RPA cases. D.O.H: days of hospitalization, IO: intraoral, TO: transaoral, TC: transcervical, RPA: retropharyngeal abscess, LA: local anesthesia, GA: general anesthesia, PARA: parapharyngeal abscess.

	Sex	Age	D.O.H	Clinical Symptoms	Microbiology	Imaging about RPA extension		Surgical treatment
1	M	64	26	Dysphagia, Odynophagia, Neck swelling, Fever	Candida glabrata		From the hyoid level to the upper mediastinum along the paraesophageal space.	TO drainage of RPA (GA) + fish bone removal
2	M	65	7	Dysphagia, Odynophagia, Fever	Staph. aureus		-	TO drainage of RPA (GA)
3	M	52	15	Dysphagia, Neck swelling, Fever	Enterococcus faecium		To the parapharyngeal space.	TO drainage of RPA (GA) + PARA
4	M	61	20	Dysphagia, Hoarseness, Neck and lumbar pain, Neck swelling, Fever	Staph. aureus, Actinomyces, Pseudomonas aeruginosa		From the hyoid to the right parapharyngeal, preepiglottic, and hypopharyngeal spaces.	TO drainage of RPA (GA) + TC incision and drainage of multiple abscesses + tracheostomy
5	M	35	15	Dysphagia, Odynophagia, Fever, Shortness of breath	No growth		To the supraglottic space.	TO drainage of RPA (GA) + tracheostomy
6	F	20	5	Dysphagia, Odynophagia, Fever	Veillonella rogosae, Staph. parasanguinis, Staph. epidermidis		To the parapharyngeal and peritonsillar space.	TO drainage of RPA (GA) + tonsillectomy + PARA
7	F	45	5	Dysphagia, Odynophagia, Fever	Candida albicans		To the prevertebral space.	TO drainage of RPA (LA)
8	F	57	11	Dysphagia, Odynophagia, Neck and lumbar pain, Neck swelling, Fever	Staph. aureus		To the prevertebral space, posterior to the posterior pharyngeal space, along the length of danger space, cervical spondylodiscitis.	TO drainage of RPA (GA) + TC incision and drainage of PARA
9	F	66	4	Dysphagia, Odynophagia, Neck and lumbar pain, Neck swelling, Fever, Shortness of breath	No growth		To the left parapharyngeal space.	Automatic IO drainage of RPA
10	M	77	42	Dysphagia, Hoarseness, Neck and lumbar pain, Neck swelling, Fever	Staph. aureus		With cervical epidural abscess, vertebral distortion, left jugular vein thrombosis, C2–C3 osteomyelitis, and prevertebral/epidural extension causing spinal cord compression.	TO drainage of RPA (LA)
11	M	47	6	Dysphagia, Fever	Staph. parasanguinis, Staph. epidermidis		-	TO drainage of RPA (LA)

## Discussion

Recent studies report an increasing incidence of RPA among adults and emphasize its more aggressive behavior [4]. Our findings are consistent with this trend, with nine out of 11 patients already demonstrating extension into adjacent deep neck spaces at the time of admission. In our series, the most common site of extension was the parapharyngeal space (patients 2, 4, 6, 8, 9), followed by prevertebral involvement (patients 7, 8, 10). Additional patterns included spread into the upper mediastinum and paraesophageal region (patient 1), as well as lateral or anterior extension into the peritonsillar space (patient 6), pre-epiglottic space (patient 4), supraglottic region (patient 5), and the danger space (patient 8). More severe cases involved postoperative cervical spondylodiscitis, and complications such as cervical epidural abscess, jugular vein thrombosis, vertebral osteomyelitis, and spinal cord compression (patients 8 and 10). These findings reflect the anatomical continuity of the retropharyngeal space, which extends from the skull base to the mediastinum, allowing direct inferior spread of infection. It is bordered anteriorly by the buccopharyngeal fascia and posteriorly divided by the alar fascia into the true retropharyngeal space and the danger space, the latter providing an uninterrupted route into the thorax and predisposing to mediastinitis. Laterally, the carotid sheath forms the boundary of the space and facilitates potential spread toward the parapharyngeal and carotid regions [1,11,12].

In this series, imaging demonstrated wide variability in the extent of disease spread. Accordingly, imaging is essential both for confirming the diagnosis and for accurately defining the involved anatomical spaces. Contrast-enhanced CT remains the gold standard for detecting abscess formation and deep neck space involvement and should be performed in all patients, whereas MRI is reserved for suspected skull base or prevertebral extension and was used in the follow-up of patients 8 and 10. The only situation in which imaging may be deferred is when securing the airway takes priority. In our series, tracheostomy was required in two patients - a slightly higher proportion than that reported by Qureshi et al., who found that 1.5% of RPA patients and 7.1% of those with parapharyngeal abscess required endotracheal intubation [11]. Differences in reported tracheostomy rates across studies may be explained by variations in study populations and disease distribution. While some reports include a heterogeneous spectrum of deep neck space infections, RPA represented only a minority of cases in certain comparative series [13]. In contrast, the present study focuses exclusively on adult patients with radiologically confirmed RPA, in whom early imaging, prompt surgical drainage, and careful airway assessment allowed safe orotracheal intubation in most cases.

Regarding aetiology, 10 out of 11 patients in our series developed spontaneous RPA, consistent with the observations of other authors [14]. This contrasts with studies in which trauma- or foreign-body-related RPAs represent the predominant cause [3,15-18]. Only one patient in our cohort (patient 1) presented with a fish bone, accounting for the single trauma-related case.

In our series, Staphylococcus species were the most frequently isolated pathogens, with additional organisms including Candida species, Enterococcus, Actinomyces, and Pseudomonas aeruginosa. These findings differ somewhat from those reported by Brook et al., who noted that the predominant anaerobic pathogens in RPA are Prevotella, Porphyromonas, Fusobacterium, and Peptostreptococcus species, while common aerobic organisms include Streptococcus pyogenes, Staphylococcus aureus, and Haemophilus influenzae [5].

The management of RPA remains a subject of ongoing debate. Although all patients require broad-spectrum intravenous antibiotics, the optimal timing and method of surgical drainage are still controversial. Several authors advocate early surgical intervention, especially in cases with airway compromise, multiloculated abscesses, or extension beyond the retropharyngeal space [19]. This approach aligns with our own clinical experience, as all patients in our series underwent drainage and survived without additional complications. This is an important outcome given the potentially fatal nature of RPA, with Konishi et al. reporting a 1.1% mortality among 1,882 cases in Japan [20] and Yang et al. documenting 115 deaths among 5,779 patients in Taiwan [4]. Surgical drainage may be performed via transoral, transcervical, or combined approaches, depending on the location and the pattern of spread. On the other hand, others report favorable outcomes with conservative medical therapy in selected patients [21]. Overall, treatment strategies were tailored to disease severity, anatomical involvement, airway risk, and culture results.

Limitations

The main limitations of this study include its retrospective design, small sample size, and single-center setting, which limit statistical power and generalizability. Variability in clinical decision-making and prior antibiotic use may have influenced management strategies and microbiological results. In addition, the lack of standardized long-term follow-up precluded assessment of delayed complications.

## Conclusions

This study underscores the severity and complexity of RPA in adults, particularly with regard to radiological patterns of spread and their implications for airway management and surgical decision-making. Most patients presented with extension into adjacent deep neck spaces. The anatomical continuity of the retropharyngeal space facilitates rapid spread to the parapharyngeal, prevertebral, and mediastinal regions, underscoring the need for early recognition and prompt intervention. Contrast-enhanced CT is essential for diagnosis and for defining the extent of involvement, while airway evaluation must always take priority. Microbiological analysis revealed a predominance of Staphylococcus species, with additional polymicrobial infections in several cases. Broad-spectrum intravenous antibiotics and early surgical drainage remain the cornerstones of treatment.
